# Altered TGFβ/SMAD Signaling in Human and Rat Models of Pulmonary Hypertension: An Old Target Needs Attention

**DOI:** 10.3390/cells10010084

**Published:** 2021-01-06

**Authors:** Takayuki Jujo Sanada, Xiao-Qing Sun, Chris Happé, Christophe Guignabert, Ly Tu, Ingrid Schalij, Harm-Jan Bogaard, Marie-José Goumans, Kondababu Kurakula

**Affiliations:** 1 Amsterdam UMC, Department of Pulmonology, Amsterdam Cardiovascular Sciences, Vrije Universiteit Amsterdam, 1081 HV Amsterdam, The Netherlands; t.jujo@amsterdamumc.nl (T.J.S.); x.sun@amsterdamumc.nl (X.-Q.S.); c.happe@amsterdamumc.nl (C.H.); i.schalij@amsterdamumc.nl (I.S.); hj.bogaard@amsterdamumc.nl (H.-J.B.); 2 INSERM UMR_S 999 (Pulmonary Hypertension: Pathophysiology and Novel Therapies), Hôpital Marie Lannelongue, 92350 Le Plessis-Robinson, France; christophe.guignabert@inserm.fr (C.G.); lyieng@gmail.com (L.T.); 3 School of Medicine, Université Paris-Saclay, 94270 Le Kremlin-Bicêtre, France; 4 Laboratory for Cardiovascular Cell Biology, Department of Cell and Chemical Biology, Leiden University Medical Center, 2300 RC Leiden, The Netherlands; M.J.T.H.Goumans@lumc.nl

**Keywords:** pulmonary arterial hypertension, TGF-β signaling, SMADs, animal models of pulmonary hypertension

## Abstract

Recent translational studies highlighted the inhibition of transforming growth factor (TGF)-β signaling as a promising target to treat pulmonary arterial hypertension (PAH). However, it remains unclear whether alterations in TGF-β signaling are consistent between PAH patients and animal models. Therefore, we compared TGF-β signaling in the lungs of PAH patients and rats with experimental PAH induced by monocrotaline (MCT) or SU5416+hypoxia (SuHx). In hereditary PAH (hPAH) patients, there was a moderate increase in both TGFβR2 and pSMAD2/3 protein levels, while these were unaltered in idiopathic PAH (iPAH) patients. Protein levels of TGFβR2 and pSMAD2/3 were locally increased in the pulmonary vasculature of PAH rats under both experimental conditions. Conversely, the protein levels of TGFβR2 and pSMAD2/3 were reduced in SuHx while slightly increased in MCT. mRNA levels of plasminogen activator inhibitor (PAI)-1 were increased only in MCT animals and such an increase was not observed in SuHx rats or in iPAH and hPAH patients. In conclusion, our data demonstrate considerable discrepancies in TGFβ-SMAD signaling between iPAH and hPAH patients, as well as between patients and rats with experimental PAH.

## 1. Introduction

Pulmonary arterial hypertension (PAH) is a fatal disease characterized by elevated pulmonary arterial pressures eventually leading to right heart failure and premature death [1]. Obstructive and complex lesions in pulmonary arteries (diameter <500 μm) are pathological features of PAH [2,3]. Accumulations of pulmonary arterial smooth muscle cells (PASMCs) and endothelial cells (PAECs) within the remodeled pulmonary arterioles contribute to progressive luminal narrowing [4]. Despite substantial progress in the treatment of PAH, the disease remains incurable and life-threatening [1]. There is an urgent need to understand the precise mechanisms of this disease to enable the identification of novel targets and promising drug candidates.

Transforming growth factor (TGF)-β signaling plays a crucial role in the pathogenesis of PAH [4]. Increased TGF-β1 levels in plasma and PASMCs from PAH patients have been reported [5,6]. Unbalanced TGF-β signaling induces proliferation in PAECs, PASMCs and fibroblasts, and leads to inflammation, endothelial to mesenchymal transition and fibrosis [7,8,9,10]. TGF-β signaling is considered as a potential target for PAH treatment. The kinase inhibition of TGF-β receptor 1 (TGFβR1) suppressed the abnormal proliferation of PASMCs and PAECs and improved hemodynamics and vascular remodeling in PAH animal models [11,12,13]. Sotatercept is a ligand trap with high selectivity for multiple proteins within the Tβ superfamily, including TGF-β1-3, activins, GDFs, and others, and thereby blocking TGF-β signaling. Sotatercept decreased the expression of plasminogen activator inhibitor (PAI)-1, a known target gene of the TGF-β pathway and suppressed the aggravation of hemodynamics and vascular remodeling in experimental animal models of PAH [14]. Based on the results of preclinical studies, a clinical trial of sotatercept in PAH patients has been performed (PULSAR study). The authors report that sotatercept significantly improved pulmonary vascular resistance and 6-minute walk distance in PAH patients [15], although the extent of alterations in TGF-β signaling by sotatercept was not completely known. Thus, despite several previous studies, the impact of TGF-β signaling on PAH in patients and animal models is still unclear. Therefore, it is important to understand this signaling in both PAH patients and animal models in detail.

The monocrotaline (MCT) and sugen–hypoxia (SuHx) rat models are two established animal models for PAH preclinical research. MCT rats receive a single injection of MCT, an alkaloid derived from the seeds of *Crotalaria spectabilis* [16]. The injected MCT is metabolized in the liver to its active form, inducing damage in the pulmonary circulation [17]. The SuHx model depends on a single injection of the vascular endothelial growth factor receptor inhibitor Sugen (SU5416) to induce EC apoptosis combined with 10% hypoxia for 3–4 weeks [18]. In the two animal models, pulmonary vascular remodeling and right ventricle (RV) hypertrophy develop with some resemblance to the pathological changes seen in human PAH [19,20]. However, even those well established animal models do not completely reflect the pathogenesis of human PAH. Both models rely on chemically and hypoxic induced damage/stress to initiate development of PAH in the following weeks, while in the clinical situation, PAH progresses much less rapidly. Moreover, inflammatory and genetic factors associated with the development of PAH [3] are different between humans and animals. Therefore, it is reasonable to speculate that TGF-β signaling in PAH animal models is different than in PAH patients.

Previous pre-clinical PAH studies showed controversial data with respect to the TGF-β signaling pathway [4]. A study in MCT rats demonstrated downregulated protein levels of TGFβR1, TGFβR2, SMAD3, and SMAD4 and the reduced phosphorylation of SMAD2 [21]. Conversely, other studies reported that TGF-β signaling was upregulated in MCT and chronic hypoxia rodent models of PAH, demonstrated by increased TGF-β1 levels in plasma and the increased phosphorylation of SMAD3 [11,13,14,22,23,24]. Thus, existing information on TGF-β signaling in PAH patients and animal models is limited and controversial. Clarifying the similarities and differences in TGF-β signaling in PAH patients and animal models may help to better understand the role of TGF-β signaling in PAH and may provide valuable information for the development of future therapies focused on balancing TGF-β/BMP signaling.

The purpose of this study is to provide an overview of key players in canonical TGF-β-SMAD2/3 signaling in PAH patients and PAH animal models, and to provide the basic characterization information necessary for future translational research. TGF-β receptors and downstream SMAD signaling in the lungs and pulmonary vasculature of PAH patients and rats with experimental PAH were investigated by immunofluorescence. The characteristics of TGF-β signaling in whole lung extracts was determined by Western blotting. Findings related to altered TGF-β signaling were compared between patients with idiopathic and hereditary PAH (iPAH and hPAH), and between patients and animal models.

## 2. Materials and Methods

### 2.1. Collecting Human Tissue Samples

The investigations using human lung samples were approved by the Institutional Review Board of the VU University Medical Center (Approval number: VUMC BUP 2013-5B) and Comité de Protection des Personnes (CPP) Ile-de-France VII, Paris (Approval number: 2018-A01252-53). In this study, autopsied lung samples were derived from iPAH (n = 7) and hPAH patients (n = 7), and control patients (n = 7) who died from other non-pulmonary cause according to the method of our previous report [25].

### 2.2. Preparing PAH Animal Models

Animal experiments were approved by the institutional Animal Care and Use Committee of the VU University, Amsterdam, The Netherlands, and were conducted according to the European convention for the protection of the vertebrate animals used for experimental and other specific purposes. All animals were kept in standard conditions (22 °C, and a 12 h light/dark cycle). All rats had free access to food and filtered water. In this study, we used two types of PAH models, i.e., SuHx and MCT rats. The SuHx and MCT rat models were prepared based on the methods described previously [26,27]. As briefly, 25 mg/kg of SU5416 (weight 130 g to 200 g, Tocris Bioscience, #3037, Bristol, United Kingdom) dissolved in carboxymethylcellulose (CMC) were subcutaneously injected into Sprague Dawley rats (n = 6, Charles River, Sulzfeld, Germany). The rats were kept in 10% O_2_ for 4 weeks and were followed by housing in normoxia for 6 weeks. MCT animals were prepared by a single subcutaneous injection of MCT 60 mg/kg dissolved in sterile saline (MCT, Sigma-Aldrich, Zwijndrecht, The Netherlands) to Wistar rats (n = 6, weight 125 to 150 g, Envigo, Horst, The Netherlands) and followed by the exposure of normoxia for 4 weeks (or when animals manifested signs of right heart failure). Control animals for SuHx (n = 5) or MCT rats (n = 4) received a single injection of CMC or saline. Animals were sacrificed under over sedation and the organs were resected. Saline containing 0.5% of agarose was injected through the airways at a constant pressure of 25 mmHg.

### 2.3. Reagents

Antibodies used for immunohistochemistry were shown as follows, TGFβR1 (1:100, rabbit, sc-398, Santa Cruz Biotechnology, Dallas, TX, USA), TGFβR2 (1:25, mouse, sc-17799, Santa Cruz Biotechnology, Dallas, TX, USA) and pSMAD2/3 (1:50, goat, sc-11769, Santa Cruz Biotechnology, Dallas, TX, USA) followed by AlexaFluor 488 donkey-anti-rabbit (1:200, A21206, Invitrogen, Waltham, MA, USA), AlexaFluor 647 donkey-anti-mouse (1:200, A31571, Invitrogen, Waltham, MA, USA) or AlexaFluor 488 donkey-anti-goat (1:200, A11055, Invitrogen, Waltham, MA, USA), respectively; pre-conjugated anti-actin α-smooth-muscle-actin–Cy3 (α-SMA, C6198, Sigma, St. Louis, MO, USA). Antibodies for Western blot analysis were shown as follows: rabbit anti-vinculin (1:500, sc-5573, Santa Cruz Biotechnology, Dallas, TX, USA); rabbit anti-phospho-SMAD 2/3 (1:1000, #8828, Cell Signaling, Danvers, MA, USA); rabbit anti-SMAD2/3 (1:1000, sc-398, Cell Signaling, Danvers, MA, USA); rabbit anti-TGFβR1 (1:500, sc398, Santa Cruz Biotechnology, Dallas, TX, USA); mouse anti-TGFβR2 (1:500, sc-17799, Santa Cruz Biotechnology, Dallas, TX, USA); anti-mouse IgG HRP-conjugated antibodies (1:4000, P0447, Dako, Jena, Germany); and anti-rabbit IgG HRP-conjugated antibodies (1:4000, P0448, Dako, Jena, Germany).

### 2.4. Immunofluorescence Staining and Quantification Analysis

Lung tissues were fixed in formalin, embedded in paraffin, and sliced 4 μm thick. After deparaffinizing and washing, pathological slides were incubated with antigen unmasking solution (H330, Vector laboratories, Burlingame, the United Kingdom) for 20 min for antigen retrieval. The slides were blocked with 1% bovine serum albumin for 90 min, which was followed by incubation with primary antibodies at 4 °C overnight. The slides were incubated with secondary antibodies at room temperature for 1 h. Counter staining was performed using 4′6-diamidino-2-phenylindole (DAPI, H-1200, Vector Labs). Image collection and quantification were performed as described in our previous study [27]. Briefly, the images were collected using a ZEISS Axiovert 200M Marianas inverted microscope at ×400 and a digital microscope software (Slidebook 6, Intelligent imaging innovations). For evaluating the protein expressions, the fluorescent intensity of each vessel was measured within the area co-stained with α-SMA. More than 20 vessels per section were measured.

### 2.5. Western Blot Analysis

For isolating the protein, resected organs were homogenized using RIPA buffer (Sigma-Aldrich) containing protease and phosphatase inhibitors. The concentration of extracted protein was determined using the Pierce 660 nm protein assay kit (Thermo Scientific, Waltham, MA, USA). The protein lysates were separated by SDS-PAGE and transferred to nitrocellulose membranes. The membranes were blocked with 5% bovine serum albumin in Tris-buffered saline containing Tween-20 (TBS-T) at room temperature for 1 h. The blocked membranes were incubated with primary antibodies at 4°C overnight. After washing membranes with TBS-T, the membranes were incubated with secondary antibodies at room temperature for 1 h. Each protein was detected by chemiluminescence using ECL Prime Western Blotting Detection Reagent (GE, Chicago, IL, USA) and Amersham Imager 600 (GE).

### 2.6. Isolation of mRNA and qPCR

mRNA isolation and qPCR analysis were performed as described previously [28]. The primer sequences will be provided upon request.

### 2.7. Measurement of TGF-β1 by ELISA

The levels of TGF-β1 were measured in plasma samples from control, MCT and SuHx rats using a TGF-β1 ELISA kit (Rat LAP TGF beta 1 Ready-SET-Go, 88-50680-22, eBioscience) and followed the protocol provided by the manufacturer.

### 2.8. Statistical Analysis

All data were analyzed using a statistical software, GraphPad Prism (ver. 8, San Diego, CA, USA) in a blinded manner. Continuous variables were described as the mean ± SD. Normality of data was checked and either log-transformation or a non-parametric test was performed if the data were not normally distributed. Unpaired Student’s t-tests were used for comparisons between the two groups. Multiple comparisons were assessed by one-way ANOVA, followed by Dunnett’s post-hoc test. A *p*-value <0.05 was considered significant.

## 3. Results

### 3.1. Increased TGF-β1 in PAH Animal Models

**Figure 1:**
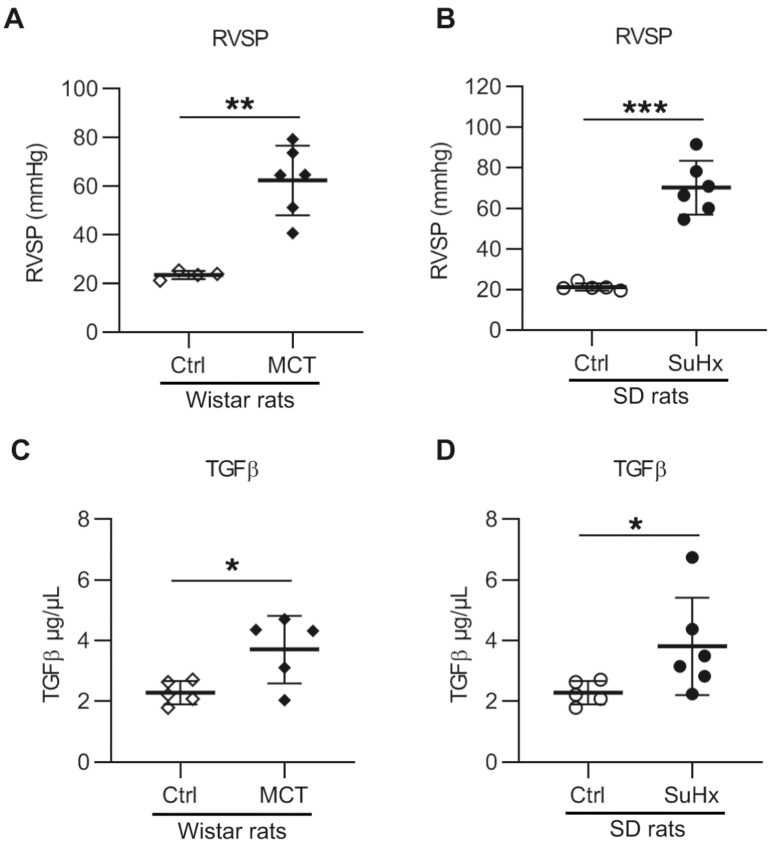
Right ventricle (RV) systolic pressure and serum transforming growth factor-β 1 (TGF-β1) levels in animal models with pulmonary arterial hypertension: (**A**) RV systolic pressure (RVSP) in monocrotaline (MCT) rats. n(Ctrl) = 4, n(MCT) = 6; (**B**) RVSP in sugen–hypoxia (SuHx) rats. n(Ctrl) = 5, n(SuHx) = 6; (**C**) serum TGF-β1 level in MCT rats. n(Ctrl) = 5, n(MCT) = 5; (**D**) serum TGF-β1 level in SuHx rats. n(Ctrl) = 5, n(SuHx) = 6. * *p* < 0.05, ** *p* < 0.01, *** *p* < 0.001.

Increased RV systolic pressure (RVSP) was observed in both MCT and SuHx rats (62 ± 14 mmHg and 70 ± 13 mmHg, respectively, see Figure 1A,B). Since previous studies revealed increased plasma levels of TGF-β1 in both iPAH and hPAH patients, we measured the levels of TGF-β1 protein in the plasma of PAH animal models. Consistent with the findings in PAH patients [5], we found that the TGF-β1 protein levels in plasma were significantly increased in both MCT and SuHx rats (Figure 1C,D). Furthermore, there was no significant correlation between the increased RVSP and plasma TGF-β1 level in either of the rat models (data not shown).

### 3.2. Increased Levels of TGFβR2 in Pulmonary Vasculature of Animal PAH Models

**Figure 2:**
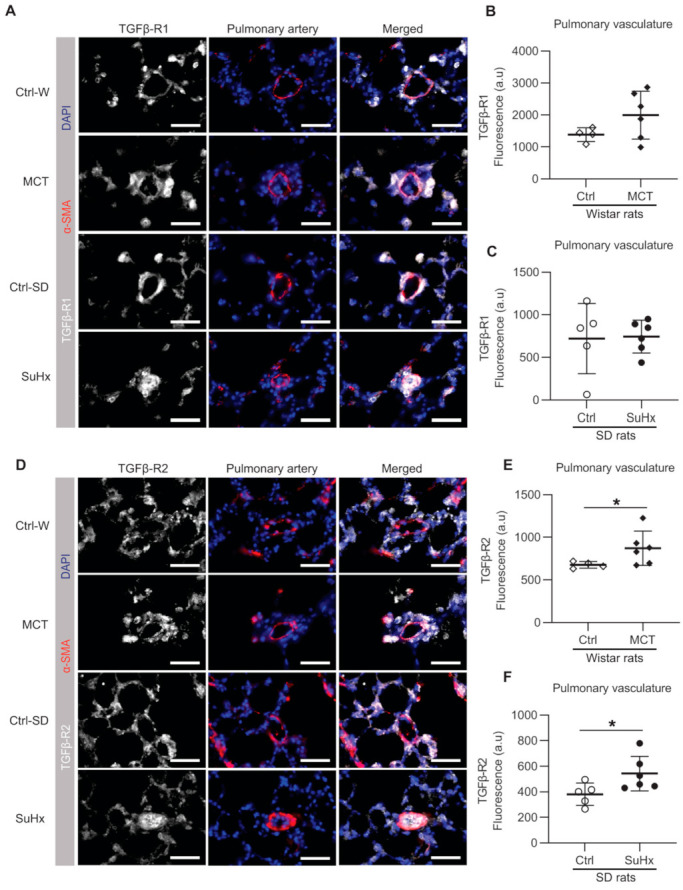
Immunofluorescence in pulmonary vasculature. (**A**) Representative images of immunofluorescence for transforming growth factor-β receptor 1 (TGFβR1); white = TGFβR1; red = α-SMA; blue = nuclei. (**B**,**C**) show the quantification data of TGFβR1 expression within the area co-stained with α-SMA; (**D**) representative immunofluorescent images for TGFβR2; white = TGFβR2; red = α-SMA; blue = nuclei; (**E**,**F**) shows the quantification data of TGFβR2 expression within the area co-stained with α-SMA. Ctrl: control rats, MCT: monocrotaline rats, SuHx: sugen–hypoxia rats, SD rats: Sprague Dawley rats, α-SMA=alpha smooth muscle actin. Scale bar = 50 μm. Wistar rats: n(ctrl) = 4, n(MCT) = 6. SD rats: n(ctrl) = 5, n(SuHx) = 6. * *p* < 0.05.

Then, we investigated the local expression pattern of TGF-β receptors in the lung vasculature patients and rats by immunofluorescence staining. The lung vascular expression of TGFβR1 in MCT and SuHx rats did not differ from their respective controls (Figure 2A–C). However, the expression of TGFβR2 was significantly increased in the pulmonary vasculature of both experimental PAH models (Figure 2D–F).

### 3.3. Differential Expressions Levels of TGF-β Receptors in Lungs of PAH Patients and PAH Animal Models

**Figure 3:**
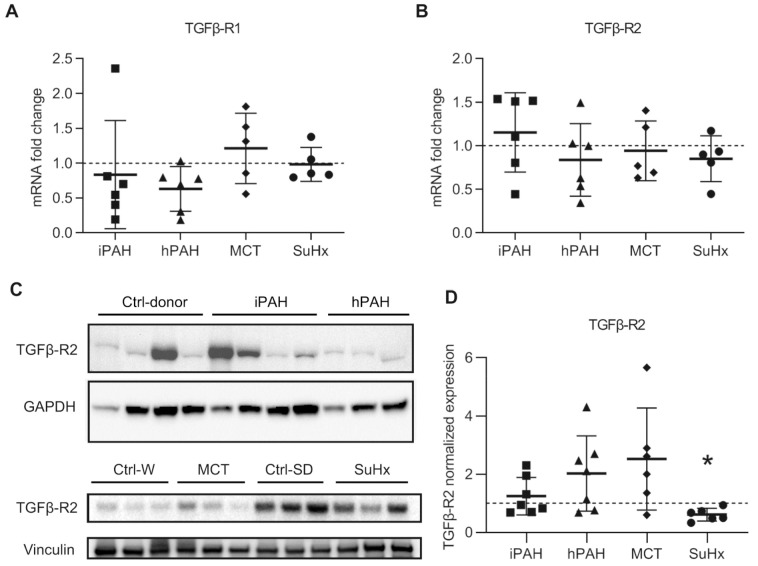
The expression of transforming growth factor-β receptors (TGFβR): (**A**) the mRNA expression of *TGFβR1* analyzed by qPCR; (**B**) the mRNA expression of *TGFβR2* analyzed by qPCR. n(iPAH) = 6, n(hPAH) = 6, n(MCT) = 5, n(SuHx) = 5; (**C**) representative image of Western blot analysis in whole lung lysates; (**D**) quantification data of Western blot analysis normalized to vinculin. n(iPAH) = 7, n(hPAH) = 7, n(MCT) = 6, n(SuHx) = 6. Quantification in each group is the fold change corrected by its own control group. The control group of iPAH and hPAH: donors without PAH. The control group of MCT: healthy Wistar rats. The control group of SuHx: healthy Sprague Dawley rats. iPAH: idiopathic pulmonary arterial hypertension, hPAH: hereditary pulmonary arterial hypertension, Ctrl: control, MCT: monocrotaline rats, SuHx: sugen–hypoxia rats. * *p* < 0.05, compared to its respective control group.

To determine the expression of TGF-β receptors in whole lungs, we isolated mRNA and proteins from the whole lung lysates of iPAH and hPAH patients, and from animals with experimental PAH. mRNA expression of TGFβR1 and TGFβR2 in the total lung of iPAH and hPAH patients was not different from controls (Figure 3A,B). However, Western blot analysis using the total lung lysates showed a modest, but not significant increase in the expression of TGFβR2 protein in both groups of PAH patients (Figure 3C,D).

In animals with experimental PAH, the mRNA and protein expression of TGFβR1 was unaltered (Figure 3A,B, and Supplemental Figure S1A,B), which was consistent with the immunofluorescence data. However, the mRNA expression of TGFβR2 was unaltered in animals with experimental PAH (Figure 3A,B). Western blot analysis using total lung lysates showed a modest increase in the expression of TGFβR2 protein in MCT rats, while a significantly decrease was observed in SuHx rats (Figure 3C,D).

In summary, no clear tendencies of differential expression of TGF-β receptors between PAH patients and animal PAH models were observed.

### 3.4. No Changes in Levels of SMAD2 and SMAD4 in PAH Patients and Animal PAH Models

**Figure 4:**
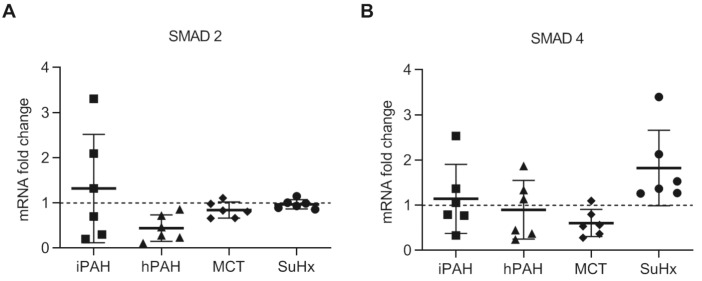
The mRNA expression of SMAD2 and SMAD4 in the whole lung lysate analyzed by qPCR: (**A**) the mRNA expression of SMAD2/3 in human PAH patients and animal PAH models; (**B**) the mRNA expression of SMAD4. n(iPAH) = 6, n(hPAH) = 6, n(MCT) = 6, n(SuHx) = 6. iPAH: idiopathic pulmonary arterial hypertension, hPAH: hereditary pulmonary arterial hypertension, Ctrl: control, MCT: monocrotaline rats, SuHx: sugen–hypoxia rats. The control group of iPAH and hPAH: donors without PAH. The control group of MCT: healthy Wistar rats. The control group of SuHx: healthy Sprague Dawley rats.

To determine downstream TGF-β signaling, we performed qPCR analyses using whole lung tissue from PAH patients, animals, and their respective controls. We found no changes in the mRNA levels of SMAD2 and SMAD4 in iPAH or hPAH, nor in the two PAH animal models (Figure 4A,B). The further analysis of the inhibitory SMADs of the TGF-β signaling cascades in PAH animal lungs revealed a reduction in the expression of SMAD7 mRNA only in MCT rats, while the expression of SMAD6 was unaltered in both rat models (Supplementary Figure S1E,F).

### 3.5. Discrapancies in pSMAD2/3 Levels in the Lungs of PAH Patients and Animal Models of PAH

**Figure 5:**
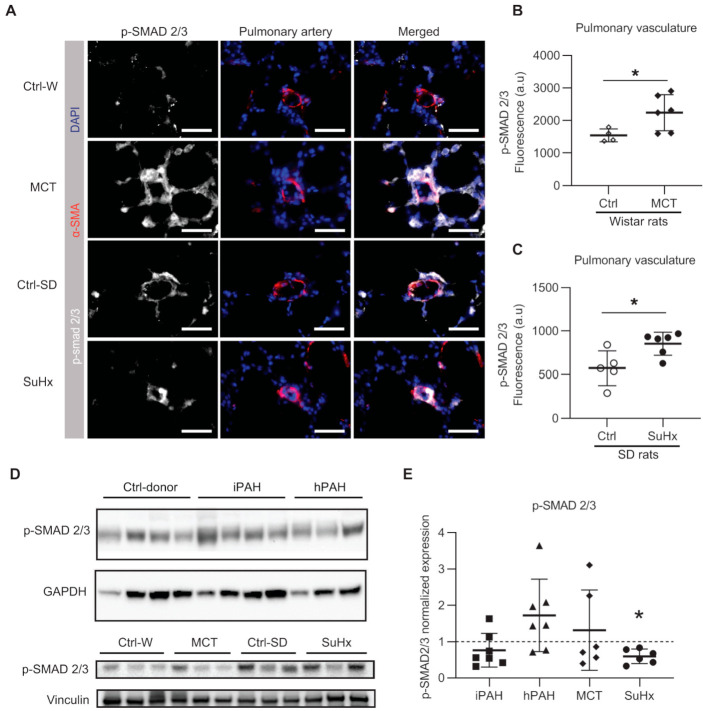
The phosphorylation of SMAD2/3 in human PAH and animal models of PAH: (**A**) representative immunofluorescent images; white = pSMAD2/3; red = α-SMA; blue = nuclei. Scale bar = 50 μm; (**B**) quantified data of pSMAD2/3 expression within the area co-stained with α-SMA in MCT rats. Wistar rats: n(ctrl) = 4, n(MCT) = 6; (**C**) quantified data of pSMAD2/3 expression within the area co-stained with α-SMA in SuHx rats. SD rats: n(ctrl) = 5, n(SuHx) = 6; (**D**) representative images of Western blot analysis in whole lung lysates. (**E**) The quantification data of phospho-SMAD (pSMAD) 2/3 expression normalized to vinculin. n(iPAH) = 7, n(hPAH) = 7, n(MCT) = 6, n(SuHx) = 6. iPAH: idiopathic pulmonary arterial hypertension, hPAH: hereditary pulmonary arterial hypertension, Ctrl: control, MCT: monocrotaline rats, SuHx: sugen–hypoxia rats. * *p* < 0.05.

Since the expression levels of SMAD2 and SMAD4 were unchanged, we then determined the levels of pSMAD2/3 as a read-out for the activation of the TGF-β signaling pathway. Immunofluorescent staining showed that the levels of pSMAD2/3 was significantly increased in the pulmonary vasculature of both MCT and SuHx rat models (Figure 5A–C).

Western blot analysis showed no difference in the levels of pSMAD2/3 in iPAH and hPAH patients (Figure 5D,E). Interestingly, while Western blot analysis showed a moderate increased level of pSMAD2/3 in MCT rats, a significant decrease in pSMAD2/3 was observed in SuHx rats (Figure 5D,E) when compared to their respective controls. In MCT rats, an increased ratio of pSMAD2/3 to total SMAD2/3 (tSMAD2/3) was found, which was accompanied by a reduction in t-SMAD2/3 (Supplementary Figure S1A,C,D).

### 3.6. Plasminogen Activator Inhibitor-1 in Lungs in PAH Patients and Animal PAH Rats

**Figure 6:**
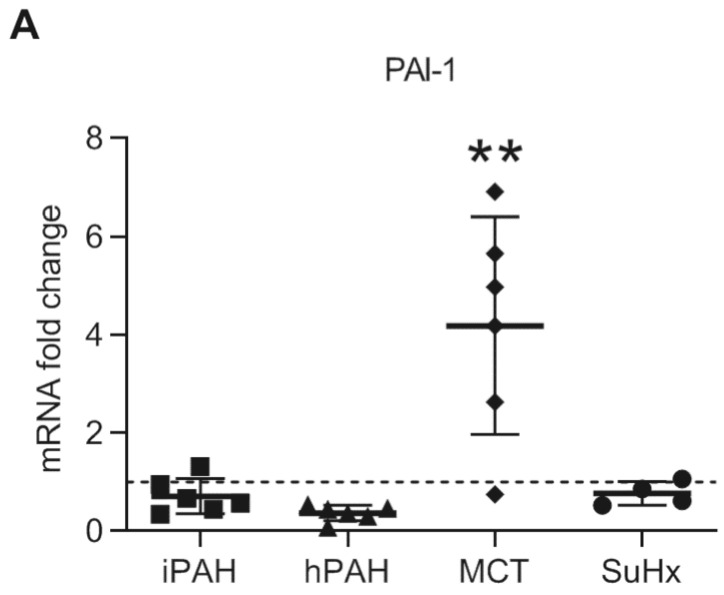
The mRNA expression of plasminogen activator inhibitor-1 in whole lung lysates was analyzed by qPCR. n(iPAH) = 6, n(hPAH) = 6, n(MCT) = 6, n(SuHx) = 4. ** *p* < 0.01.

To further determine downstream TGF-β signaling, we analyzed the mRNA levels of PAI-1, a known target gene of the TGF-β/TGFβR1/Smad2/3 pathway [4]. Interestingly, while the expression of PAI-1 was unaltered in iPAH and hPAH patients (Figure 6), the expression of PAI-1 was significantly increased in MCT rats while no change was observed in in SuHx rats (Figure 6).

## 4. Discussion

The present study describes the expression levels of TGF-β receptors and their direct downstream signaling component pSMAD2/3 in lung tissue of PAH patients as well as in two established rat models of PAH (MCT and SuHx). Moreover, the expression patterns were investigated more specifically in the pulmonary vasculature of the two PAH rat models. In this study, we report for the first time increased canonical TGF-β signaling in SuHx rats. We confirmed an elevation of serum TGF-β1 and increased the local expressions of TGFβR2 and p-SMAD2/3 in the pulmonary vasculature both in MCT and SuHx rats. Conversely, differential expression of TGF-β/SMAD signaling was found between pulmonary vasculature and whole lung, between different animal models, and between PAH patients and the animal models. These findings suggest that the careful interpretation of the data on TGF-β/SMAD signaling in whole lung lysates and pulmonary vasculature is necessary to evaluate and interpret the outcome of preclinical studies.

### 4.1. Altered TGFβ-SMAD2/3 Signaling in Experimentally Induced PAH Animal Models

Previous studies reported that levels of TGF-β1 are markedly increased in several PAH animal models [29]. In the present study, we confirmed the increased plasma level of TGF-β1 protein in both MCT and SuHx rats. Of note, this observed increase is independent of the strain of rats (Wistar rats for MCT and Sprague Dawley rats for SuHx). Previous studies demonstrated the increased expression of TGF-β1 in lungs of MCT and SuHx rat models, regardless of Wistar [30] or Sprague Dawley rats [13,29], which supported our results.

However, previous studies showed controversial results regarding TGF-β receptors in the lungs of MCT rats. Long et al. reported increased mRNA expression of TGFβR2 in whole lung lysates of MCT rats on day 21 [13]. On the other hand, Zakrzewicz et al. described opposite results with reduced mRNA and protein expression of TGFβR2 in whole lung lysates of MCT rats at day 28, while it was unaltered at day 14 [21]. In the current study, we found a moderate increase in TGFβR2 protein expression in the whole lung lysates in MCT rats between day 18 to 25 (sacrificed when rats manifest signs of right heart failure), while mRNA levels of TGFβR2 did not differ from those of control rats. The controversial results from different studies in MCT rats may be due to protocol differences in MCT experiments, especially with regard to duration and animal strains. Of note, MCT experiments were performed using Sprague Dawley rats in some previous studies, while Wistar rats were used for MCT experiments in the current study. We found that the protein expression of TGFβR2 in lungs in untreated Wister rats was significantly lower than that in untreated Sprague Dawley rats (data not shown). Therefore, the expression of TGFβR2 in the lungs of the experimental PAH models can be affected by the strain of rats, which needs to be considered for future translational studies.

The phosphorylation of SMAD2/3 and the mRNA expression of PAI-1 was increased in MCT rats in the current study. Previous studies demonstrated that the levels of pSMAD2/3 and PAI-1 are augmented in MCT rats [12,13], which supports our results. The total levels of SMAD2/3 were greatly reduced in MCT in this study. A decrease in SMAD3 expression in the lungs of MCT rats was described previously [21,29], consistent with our results. It was speculated that chronic TGF-β exposure may cause decreased SMAD3 expression in the lungs of MCT rats, although the precise mechanism remains unclear [29]. It has to be noted that the expression of p-SMAD2/3 was not reduced accordingly, despite a reduced level of t-SMAD2/3, resulting an increase in p-SMAD2/3 to t-SMAD2/3 ratio in MCT rats. Together with the observation of a moderate increased TGFβR2 and decrease in SMAD7, which is an inhibitory SMAD, these results clearly reveal an upregulation mechanism of TGF-β signaling in the lungs of MCT rats. Consistent with the trends shown by Western blot analysis on whole lung lysates, the protein expressions of TGFβR2 and pSMAD2/3 were markedly increased in the pulmonary vasculature of MCT rats compared to the controls.

We showed for the first time a significant increase in the protein levels of TGFβR2 and pSMAD2/3 in the lung vasculature of SuHx rats. Interestingly, the protein expressions of TGFβR2 and p-SMAD2/3 in whole lung lysates are reduced in SuHx rats. This discrepancy in expression after studying whole lung lysates vs. the lung vasculature may suggest differential TGF-β-pSMAD2/3 signaling in different cell types in the SuHx lung. Overall, together with unaltered inhibitory SMADs and PAI-1 mRNA expression in SuHx rats, these observations indicate the involvement of different regulation mechanisms and different target genes of TGF-β-SMAD2/3 signaling in the two PAH animal models. The differences may result from the different stimuli that induce PAH in these models, or from the different disease stages manifested by the two animal models.

### 4.2. Unaltered TGFβ-SMAD2/3 Signaling in the Lungs of PAH Patients

Ample evidence demonstrates the key role of TGF-β signaling in the initiation and progression of pulmonary vascular remodeling in PAH. Increased TGF-β signaling can not only lead to pro-proliferative and anti-apoptotic responses in PAECs and PASMCs, but also to the increased production of inflammatory cytokines and chemokines [31,32]. Previous studies revealed aberrant TGF-β signaling in PAH, including increased levels of TGF-β1 in serum, in the lungs and in the pulmonary vasculature of PAH patients [5,33,34]. However, in the present study, our analyses showed that there was no difference in the gene expression of TGFβR1 and TGFβR2 between iPAH, hPAH and non-PAH donors. Consistently, Western blot analysis showed no difference in protein expression of TGFβR2 and pSMAD2/3 in the lungs of iPAH patients. This is consistent with a previous study which revealed no difference in TGFβR2, SMAD2 and SMAD4 in the pulmonary vasculature of iPAH patients, but only a trend of increased p-SMAD2 [35]. Similarly, Western blot analysis showed only a modest increase in TGFβR2 and pSMAD2/3 levels in the lung tissue of hPAH patients. The slight differences between iPAH and hPAH patients may be partly explained by a previous study, which showed that PAECs expressing a mutant BMPR2 can release higher levels of TGF-β1 into the medial layer which may result in enhanced TGF-β signaling [36].

The inconsistency between increased TGF-β1 and unaltered (or slightly increased) TGF-β signaling molecules in the PAH lung might be explained by an inactive form of TGF-β1, an unmatched ligand binding and a different response to TGF-β1 in different cell types. It is possible that the levels of TGF-β signaling molecules are increased only in certain types of cells in the PAH lungs, such as the immune cells around the blood vessels, which cannot be distinguished in the whole lung lysates. Consistent with this notion, a specific TGFβR2 upregulation in pericytes has been recently identified in explanted lung tissues from PAH patients [37]. Though a previous study only showed a modest increase in pSMAD2 in the pulmonary vasculature of iPAH patients, without any difference in TGF-β receptors or SMADs, it has to be noticed that pSMAD2 expression was found to be correlated inversely with pulmonary artery size and the extent of vascular wall remodeling [35]. Therefore, the role of increased TGF-β signaling in the pathogenesis of human PAH cannot be underestimated despite the unaltered molecular signaling observed in this study. Further studies need to be performed to investigate the expression pattern of TGF-β signaling molecules in different sizes and grades of occluded pulmonary vessels and different cell types in PAH lungs.

### 4.3. Differential Regulation of TGF-β-SMAD2/3 Signaling Levels in PAH Patients and Animal PAH Models

Our findings did not only reveal differences in TGF-β-SMAD2/3 signaling between MCT and SuHx PAH models, but also revealed discrepancies between PAH patients and PAH animal models. TGF-β1 is a pluripotent cytokine that mediates its effects through a receptor composed of TGFβR2 and TGFβR1. Upon the binding of TGF-β, TGFβR2 becomes activated and recruits TGFβR1 and the resulting TGF-β receptor complex activates the SMAD signaling pathway [4]. In the present study, despite an increase in TGFβR2 levels in hPAH patients and two animal models of PAH, we did not observe an increase in TGFβR1 levels in all analyses (immunofluorescence, mRNA and protein). Therefore, it is likely that TGFβR1 is not the rate limiting factor in this equation, and the observed increase in downstream pSMAD2/3 levels predominantly resulted from increased TGFβR2 levels via the excessive binding of activated TGF-β1 present in the animal PAH models and PAH patients.

The discrepancies in TGF-β-SMAD2/3 signaling may reflect the alterations of TGF-β-SMAD2/3 signaling along the disease progression at different stages. In the current study, the human PAH lung samples are derived from autopsied PAH patients, obviously at the end stage of their disease. Conversely, the lung samples from PAH animal models were obtained 3 or 4 weeks after treatments with MCT or Su5416 injection, and those animals seem to be at earlier stages of the disease with increased TGF-β signaling. Consistent with this assumption, previous studies also revealed the differences in TGF-β signaling in the lung tissue of an MCT rat model at different time points [13,21]. If the impact of TGF-β signaling on PAH could differ between early and advanced stages, the effects of TGF-β targeted therapies including sotatercept might be different depending on the stages in PAH patients. Therefore, to promote the role of TGF-β signaling as a promising therapeutic target, further studies are needed to investigate the role of TGF-β signaling in PAH at different stages.

Our results also suggested that TGF-β signaling may play different roles in the disease progression in PAH patients and in PAH animal models. Therefore, it becomes questionable to use any of these animal models to study this signaling in PAH translational research. This confirmed the necessity in PAH translational research to use multiple PAH animal models or combine with in vitro models from PAH patients. Future studies should also focus on understanding the differences between the various cell types in the lungs since we observed the differential regulation of TGFβ-SMAD2/3 signaling in vascular cells compared to non-vascular cells in experimental PAH.

There are few limitations in the current study. Since PAH is a rare disease, the sample size was relatively small. Furthermore, the non-canonical TGF-β pathway was not investigated. Finally, compartment-specific expression studies in pulmonary vasculature with laser capture microdissections was not performed to acknowledge cell type specific responses. Therefore, future studies are warranted to delineate this signaling pathway in detail using a large number of samples and novel high-resolution technologies.

In conclusion, our study reveals a discrepancy of TGF-β-SMAD2/3 signaling in the lungs between PAH patients and PAH animal models (MCT and SuHx rat models). The careful interpretation of the pre-clinical data is necessary to evaluate the expression of TGF-β-SMAD2/3 signaling, even though the altered signaling is observed.

## Data Availability

The data presented in this study are available on request from the corresponding author due to restrictions.

## Supplementary Materials

The following are available online at https://www.mdpi.com/2073-4409/10/1/84/s1. Figure S1: The expression of transforming growth factor-β receptor1 (TGFβ-R1) and SMADs in whole lung lysates of MCT and SuHx rats.
