# Supplementary material for: Altered TGFβ/SMAD Signaling in Human and Rat Models of Pulmonary Hypertension: An Old Target Needs Attention
Source: Cells. 2021 Jan 6;10(1):84. doi: 10.3390/cells10010084 (PMC7825543; doi:10.3390/cells10010084)
Supplement: Supplementary file 1 [file cells-10-00084-s001.pdf]

cells

MDPI

Supplementary Materials

Altered TGFβ/SMAD Signaling in Human and Rat Models of Pulmonary Hypertension: An Old Target Needs Attention

Takayuki Jujo Sanada [1,†], Xiao-Qing Sun [1,†], Chris Happé [1], Christophe Guignabert [2,3], Ly Tu [2,3], Ingrid Schalij [1], Harm-Jan Bogaard [1], Marie-José Goumans [4] and Kondababu Kurakula [4,*]

1 Amsterdam UMC, Department of Pulmonology, Amsterdam Cardiovascular Sciences, Vrije Universiteit Amsterdam, 1081 HV Amsterdam, The Netherlands; t.jujo@amsterdamumc.nl (T.J.S.); x.sun@amsterdamumc.nl (X.-Q.S.); c.happe@amsterdamumc.nl (C.H.); i.schalij@amsterdamumc.nl (I.S.); hj.bogaard@amsterdamumc.nl (H.-J.B.)

2 INSERM UMR_S 999 ((Pulmonary Hypertension: Pathophysiology and Novel Therapies), Hôpital Marie Lannelongue, 92350 Le Plessis-Robinson, France; christophe.guignabert@inserm.fr (C.G.); lyieng@gmail.com (L.T.)

3 School of Medicine, Université Paris-Saclay, 94270 Le Kremlin-Bicêtre, France

4 Laboratory for Cardiovascular Cell Biology, Department of Cell and Chemical Biology, Leiden University Medical Center, 2300 RC Leiden, The Netherlands; M.J.T.H.Goumans@lumc.nl (M-J. G.)

* Correspondence: k.b.kurakula@lumc.nl; Tel.: +31-715-269-265; Fax: +31-715-268-270

† Both authors contributed equally.

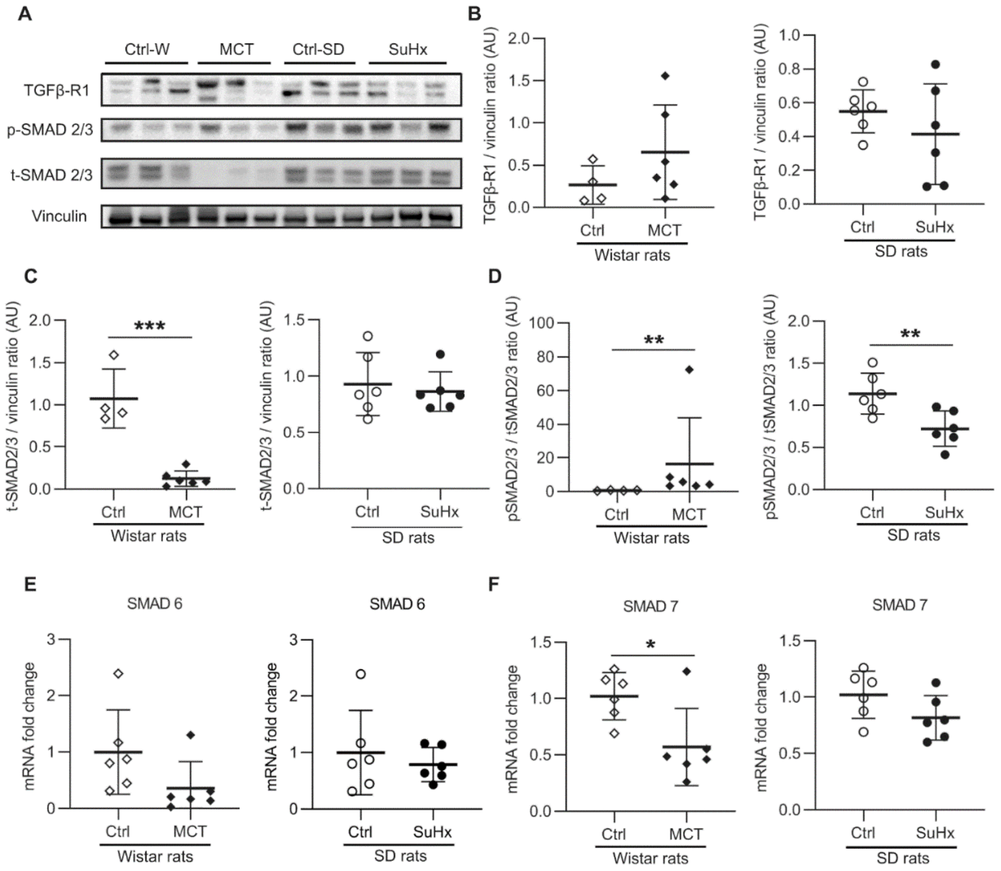

Figure 1. The expression of transforming growth factor-β receptor1 (TGFβ-R1) and SMADs in whole lung lysates of MCT and SuHx rats: (A) representative images of the Western blot analysis in whole lung lysates; (B–D) Western blot quantification data of TGFβ-R1 (B), total SMAD2/3 (t-SMAD2/3) (C), and phospho-SMAD2/3 (pSMAD2/3) (D); (E–F) mRNA expression of SMAD6 (E), and SMAD7 (F) in whole lung lysates. Ctrl: control, W: Wistar rats, SD: Sprague Dawley rats, MCT: monocrotaline rats, SuHx: sugen–hypoxia rats. *: $p < 0.05$, compared to its respective control group.
